# Metal Bezoars Causing Upper Gastrointestinal Obstruction in a Schizophrenic

**Published:** 2011-07-30

**Authors:** Zaka ur Rab Siddiqui

**Affiliations:** Department of General Surgery Unit II, Jinnah Postgraduate Medical Centre Karachi, Pakistan

## Abstract

Metal bezoars are uncommon foreign bodies (FB) in the gastrointestinal tract (GIT) and comprised of a wide variety of objects. A 17-year-old schizophrenic presented with abdominal pain and distension along with non-bilious vomiting for 2 weeks. Physical examination revealed dullness to percussion in the epigastrium. Plain radiographs revealed objects of metal density contained within a dilated stomach. Laparotomy was performed revealing metal objects in stomach.

**Keywords:** Metal bezoars, Psychiatric illness, Intestinal obstruction

**How to Cite:** Siddiqui Z. Metal bezoars causing upper gastrointestinal obstruction in a schizophrenic. APSP J Case Rep 2011; 2:14

## INTRODUCTION

A bezoar is a conglomeration of partially digested or un-digestible foreign material in the gastrointestinal tract (GIT) especially the stomach [1]. Bezoars occurring in the small intestine, colon and rectum are less common [2]. Bezoars may produce a wide variety of signs and symptoms depending upon their location, nature, volume and duration. The clinical features may range from mild pain abdomen to intestinal obstruction, perforation and peritonitis. Metal bezoars are rarely encountered in surgical practice. Only few cases have been reported in literature [3, 4]. We are reporting a case of metal bezoars in a psychiatric patient

## CASE REPORT

A 17-year-old boy presented to the accident and emergency room with mild diffuse abdominal pain and abdominal distension for two weeks, accompanied with vomiting of gastric contents along with metal fragments. The patient was under treatment for schizophrenia but not taking medications regularly. Physical examination revealed an afebrile patient with tachycardia and tachypnea. The abdomen was soft but distended. A mass was palpable in the epigastrium being dull to percussion. Plain radiograph of the abdomen showed multiple objects of metal density contained within the stomach (Fig. 1).

The patient was admitted and upper GI endoscopy was attempted to remove the FB but failed due to enormous size of bezoar. Open removal of the bezoar was then planned. At operation, a grossly dilated stomach found. Through a longitudinal gastrotomy multiple metal objects including: nails, copper wires, blade, screws, rubber bands, coins and the remains of partially digested food (measuring about half kilograms) were removed. The stomach was repaired in a double layer fashion. Rest of the GIT was normal. Postoperative recovery was uneventful. The patient was discharged and transferred to a psychiatric facility 7 days after surgery.

**Figure:**
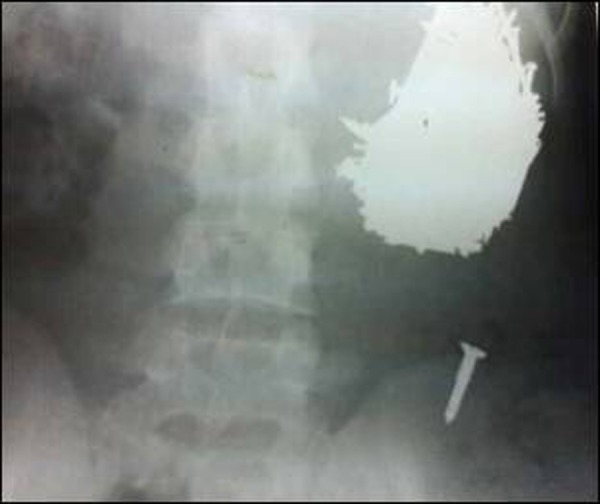
Figure 1: X-ray showing metal objects in the hugely distended stomach.

## DISCUSSION

GI bezoars account for 4% of all admissions for GIT obstruction. Approximately 10% of patients have associated psychiatric abnormalities or mental retardation therefore psychiatric evaluation and therapy are needed to prevent a recurrence. Metal bezoars are extremely rare [4, 5, 6, 7].

Metal bezoars can be easily diagnosed on abdominal radiographs as in the index case; however diagnostic difficulties arise in patients with radiolucent bezoars. GIT contrast studies and computed tomography (CT) scan are necessary in such circumstances. Upper GI endoscopy is the method of choice in detecting and dealing with esophageal, gastric and duodenal foreign bodies. Occasionally, bezoars are found incidentally when an emergency laparotomy is done secondarily to bowel obstruction [8, 9, 10].

Several treatment options can be availed for the management of gastric metal bezoars. Endoscopic retrieval is superior for small objects whereas in large objects open approach is suitable. Once the obstruction occurs, surgery is the only way to solve the problem. Frequently, synchronous bezoars are found in the stomach or other areas of the gastrointestinal tract; therefore it is mandatory to carry out a thorough exploration of the small intestine and colon to avoid future occurrence of intestinal obstruction due to a retained bezoar [8]. The recurrence has been reported in up to 14% of cases, especially in patients with psychiatric ailments [3].

## Footnotes

**Source of Support:** Nil

**Conflict of Interest:** None declared
